# A novel nomogram based on GD for predicting prognosis in hepatocellular carcinoma

**DOI:** 10.3389/fonc.2023.1174788

**Published:** 2023-11-01

**Authors:** Ying Liu, Kang Cui, Huan Zhao, Wang Ma

**Affiliations:** Department of Oncology, The First Affiliated Hospital of Zhengzhou University, Zhengzhou, Henan, China

**Keywords:** liver cancer, nomogram, glutamyltranspeptidase, prognosis liver cancer, D-dimer, prognosis

## Abstract

**Purpose:**

The prognosis of liver cancer remains unfavorable nowadays, making the search for predictive biomarkers of liver cancer prognosis of paramount importance to guide clinical diagnosis and treatment. This study was conducted to explore more prognostic markers for most HCC.

**Patients and methods:**

A total of 330 patients were enrolled in this study according to the inclusion and exclusion criteria. Follow-up data were collected for all patients until the cutoff date of the study, February 2023. In addition, patient outcomes were assessed with progression-free survival (PFS) and overall survival (OS). All statistical analysis was conducted using R 4.2.0 software.

**Results:**

Univariate analysis illustrated that the GD [the product of gamma-glutamyl transpeptidase (GGT) concentration and D-dimer concentration, GD=GGT*D-dimer] levels were related to PFS (p<0.05) and OS (p<0.05). Kaplan–Meier survival curves and log-rank tests indicated a significant difference among different levels of GD (p<0.001). Multivariate analysis demonstrated GD as an independent prognostic factor for HCC. The C-indexes of nomogram were 0.77 and 0.76 in the training or validation cohort, respectively. Area Under the Curve (AUC) of 1-, 2-, 3-, and 4-year OS showed satisfactory accuracy, and the calibration curve illustrated brilliant consistence between the ideal and predicted values.

**Conclusions:**

Herein, it was demonstrated that GD was an independent prognostic factor for HCC and revealed the potential to predict the PFS and OS in patients with HCC. Moreover, the nomogram based on GD illustrated a satisfactory prediction ability in comparison to other models without GD.

## 1 Introduction

Hepatocellular carcinoma (HCC) is the sixth most frequently occurring cancer worldwide and the third most common cause of cancer mortality (1). HCC is highly associated with chronic viral hepatitis (2, 3), alcohol (4), smoke (5), iron (6), and metabolic dysfunction (7, 8). To date, hepatitis B virus (HBV) and hepatitis C virus (HCV) have been reported as commonest risk factors for HCC. Almost half of HCCs are associated with HBV infection, and this figure is even higher in Asian. Surgical resection (9), transarterial chemoembolization (TACE) (10), ablation (11), immunotherapy (12), and targeted therapy (13) have been confirmed to be effective local or systemic therapies to improve the prognosis of liver cancer patients.

Nevertheless, the prognosis of patients with liver cancer still remains poor, making it of great clinical significance to search for the biomarkers that can accurately predict the prognosis of hepatocellular carcinoma. Alpha-fetoprotein (AFP) is the most widely used biomarker for HCC diagnosis and the evaluation of therapeutic efficacy and prognosis (14). Nevertheless, not all HCCs secret AFP, and the elevation of AFP can be observed in cirrhosis or hepatitis cases. Hence, simple and promising clinical factors for predicting the prognosis of HCC patients need further exploration. Meanwhile, hematological indicators from routine testing are not only economical but also readily available for repeated testing. Herein, efforts were made to explore laboratory biomarkers that might be useful in forecasting the prognosis in HCC. Patients with malignant tumors are prone to abnormalities in their blood coagulation and fibrinolysis systems, which can lead to elevated levels of D-dimer. Simultaneously, the elevation of gamma-glutamyl transpeptidase (GGT) is regarded as a sign of liver function damage. Quite a few studies have discovered the efficiency of GGT concentration as an indicator in tumor diagnosis and prognosis prediction (15). In this case, it was hereby speculated that GGT and D-dimer may be associated with the prognosis of HCC. As a consequence, it was found through data statistics that GGT and D-dimer had the potential to predict the PFS in HCC patients. Furthermore, GGT and D-dimer were combined to construct a novel indicator, named as GD. The GD index, short for the gamma-glutamyl transpeptidase-D-dimer Index, was defined as the product of serum GGT concentration and serum D-dimer concentration, GD=GGT*D-dimer, based on which a nomogram was built to predict the progression-free survival (PFS) and overall survival (OS) for patients with HCC. Compared with other models without GD, this predictive model manifested satisfactory predictive capacity. Thus, this nomogram was considered to have the potential to predict the PFS and OS of HCC patients, thereby providing new possibility for the prediction of the PFS and OS of HCC patients.

## 2 Materials and methods

### 2.1 Patients

A total of 921 patients with HCC were retrospectively identified from three hospital campus, The First Affiliated Hospital of Zhengzhou University (n=662), Henan Provincial Hospital (n=107), and The First Affiliated Hospital of Zhengzhou University Cancer Hospital (n=152). An additional 591 patients who did not receive TACE or were lost to follow-up were not included in our analysis. Herein, a retrospective analysis was executed based on 330 patients diagnosed with HCC from January 2018 to February 2023. We employed randomization to divide the dataset into training and validation sets at a ratio of 2:1. Patients included should meet the following criteria: 1) diagnosis of HCC, 2) patients who received TACE, 3) lesions available for evaluation, and 4) regular auxiliary examination such as imaging examination and hematological examination. Meanwhile, the main exclusion criteria of this study included 1) history of other tumors (n=15), 2) tumor surgical resection history (n=132), 3) hepatic encephalopathy that might influence examinations results (n=27), or 4) incomplete follow-up data (n=417) (**Supplementary Figure S1**). Agreement from the participants had been acquired, and participants had signed informed consent before the study and agreed to the publication of this paper. This study conformed to the Declaration of Helsinki and was approved by Ethics Committee (approval number 2022-KY-1140).

### 2.2 Data collection

Herein, several indicators, including tumor stage, age, gender, presence or absence of metastasis, presence or absence of targeted therapy, and several laboratory data were selected and incorporated. The tumor stage was classified by the Barcelona stage. Several hematology indexes of laboratory variables including alanine transaminase (ALT), aspartate transaminase (AST), GGT, blood platelet (PLT), alpha-fetoprotein (AFP), albumin, and D-dimer were retrospectively extracted from the medical records during the treatment process. Meanwhile, “x-tile app” was used to acquire the optimal cut-off points of hematological indexes with the best significance with survival analysis. Patient progression outcomes from the date of disease diagnosis to relapse or progression were assessed with PFS. OS was determined as the time from diagnosis of hepatocellular carcinoma to the date of death or the last follow-up date. Based on the guidelines of Response Evaluation Criteria in Solid Tumors (RECIST, version 1.1) (16), the response was assessed by computerized tomography (CT) and magnetic resonance imaging (MRI) every 2 months.

### 2.3 Statistical analysis

SPSS version 26.0 software and R 4.1.3 were used to implement all statistical analysis. Continuous variables were presented as medians (range), and categorical variables were shown with quantity and proportion. Continuous variables were compared by Student’s t-test, and chi-squared test was carried out to compare categorical variables, while Kaplan–Meier method was performed to compare the PFS and OS between different groups. The difference in survival curves was tested by log-rank test, and *p*<0.05 was regarded as statistically significant difference. The Cox regression analysis was conducted for multivariate analysis, and the nomogram based on Cox model was performed by rms package. In addition, the receiver operating characteristic (ROC) curves were plotted via the timeROC package, the calibration curve was plotted by the rms package, decision curve analysis (DCA) was conducted by the stdca package, and net reclassification improvement (NRI) was performed by nricens package. Additionally, integrated discrimination improvement was performed by survIDINRI package.

## 3 Results

### 3.1 Patient characteristics

Finally, 330 patients were enrolled into this study on the basis of the inclusion and exclusion criteria. Median overall survival times was 38.00 months (95% CI, 33.33–42.77), while the median progression-free survival times was 8.50 months (95% CI, 7.20–9.80) and median follow-up time was 46 months (95% CI, 40.87–51.13). The number of OS events at first, second, third, and fourth years of follow-up were 35, 84, 34, and 25 events, respectively. On the whole, 245 (74.24%) patients were male and 85 (25.76%) were female. The median age summarized in this study was 58 years (range, 28–80 years), and further clinical pathological characteristics data of the patients are shown in **Table 1**.

**Table 1:** Clinical and pathological characteristics of patients.

Characteristics	Training cohort	Validation cohort
	(n=220)	(n=110)
Age (median, range), years	56.50 (30.00–79.00)	58.50 (28.00–80.00)
Gender (male/female)	160 (72.73%) /60 (27.27%)	85 (77.27%) /25 (22.73%)
Stage (I/II/III/IV)	48 (21.81%) /67 (30.45%)/102 (46.36%)/3 (1.38%)	29 (26.36%) /40 (36.36)/40 (36.36%)/1 (0.92%)
Tumor Thrombus (with/without)	84 (38.18%) /136 (61.82%)	32 (29.09%) /78 (70.91%)
GD (median, range)	42.48 (0.42–1915.00)	44.94 (1.35–1161.54)
AFP (median, range), ng/ml	58.20 (1.58–111500.00)	39.40 (1.00–132082.00)
Size (median, range), cm	6.90 (1.00–20.00)	6.85 (12.00–22.30)
Metastasis (with/without)	188 (85.45%) /32 (14.55%)	88 (80.00%) /22 (20.00%)
Targeted therapy (with/without)	163 (74.09%) /57 (25.91%)	66 (60.00%) /44 (40.00%)
Immunotherapy (with/without)	64 (29.09%) /156 (70.91%)	28 (25.45%) /82 (74.55%)

### 3.2 Acquisition of the optimal cutoff value

In this study, GD was defined as the product of GGT concentration and D-dimer concentration, (GD=GGT*D-dimer). Then, the indicator was analyzed using the X-tile software to get the best cut-off values and grouped according to the best cut-off value. The GD value for PFS below 28.00 was classified as the low-risk group, while the GD value between 28.00 and 160.00 was classified as the median-risk group, and the GD value above 160.00 was classified as the high-risk group. The GD value for OS below 43.00 was classified as the low-risk group, while the GD value between 43.00 and 200.00 was classified as the median-risk group, and the GD value above 200.00 was classified as the high-risk group.

### 3.3 Correlation analysis among the 10 variables

In order to test the correlation of different variables, R4.1.3 corrplot package was used for correlation analysis of the filtered variables, and no significant correlation was observed among the 10 independent variables (**Supplementary Figure S2**).

### 3.4 Survival analysis

**Figure 1:**
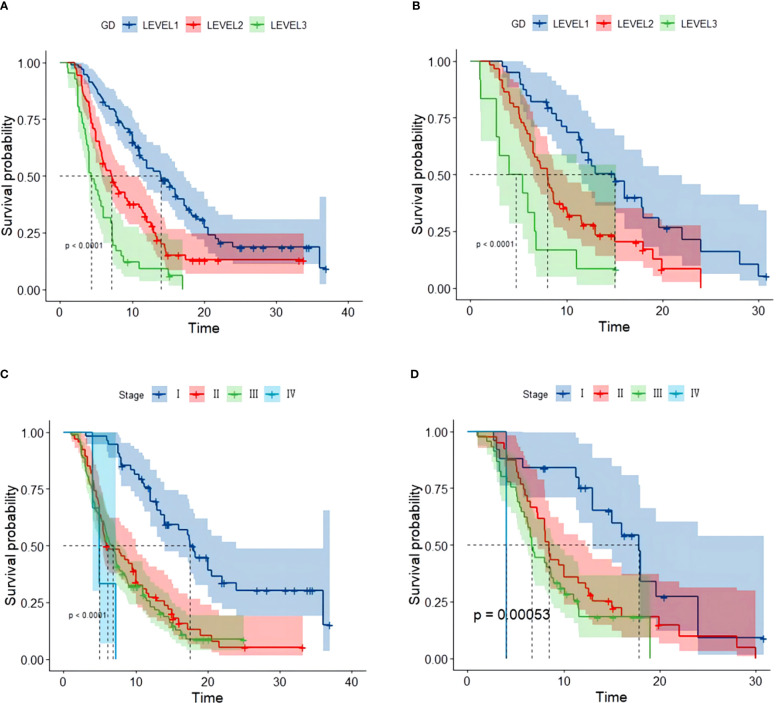
Kaplan–Meier survival curve of several biomarkers associated with PFS. **(A)** Kaplan–Meier survival curve of GD in the training and validation cohorts, **(B)** Kaplan–Meier survival curve of GD in the validation cohort, **(C)** Kaplan–Meier survival curve of stage in the training cohort, and **(D)** Kaplan–Meier survival curve of stage validation cohort.

**Figure 2:**
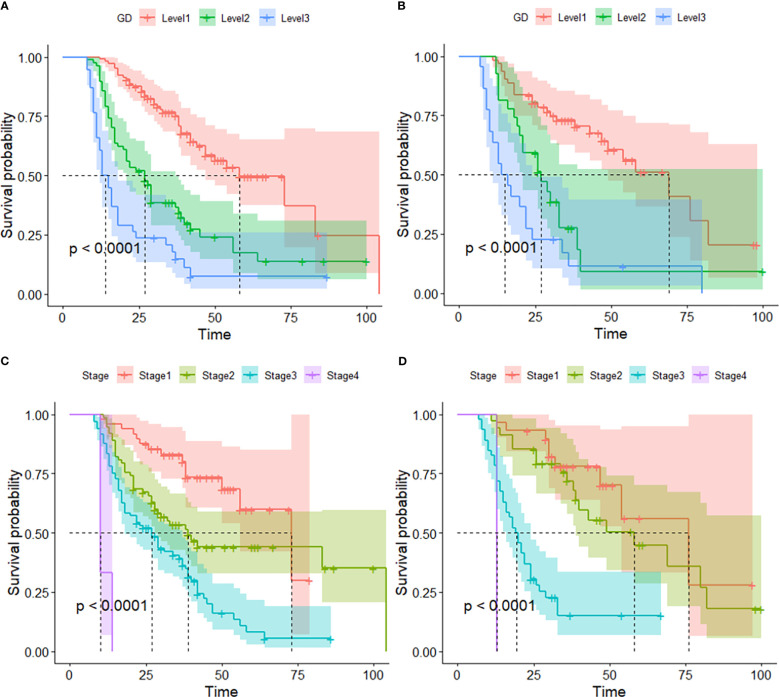
Kaplan–Meier survival curve of several biomarkers associated with OS. **(A)** Kaplan–Meier survival curve of GD in the training and validation cohorts, **(B)** Kaplan–Meier survival curve of GD in the validation cohorts, **(C)** Kaplan–Meier survival curve of Stage in the training cohort, and **(D)** Kaplan–Meier survival curve of stage validation cohort.

By the end of the cutoff date of this study, 263 (79.70%) patients had experienced disease progression per RECIST criteria, and the remaining 67 (20.30%) were manifested as disease stabilization or remission. During the study, 194 participants experienced disease-related mortality as the endpoint events. Univariate analysis was performed to confirm that single clinical pathological characteristic was connected with PFS and OS (**Table 2**). These results indicated that GD (*p*<0.001) was correlated with PFS and OS (**Table 2**), the log-rank test and Kaplan–Meier survival curves indicated that patients with lower levels of GD had longer PFS (**Figures 1A, B**) and OS (**Figures 2A, B**) than those with higher levels. Simultaneously, Log-rank tests and Kaplan-Meier survival curves indicated that patients with earlier stages had longer PFS (**Figures 1C, D**) and OS (**Figures 2C, D**). The results of multivariate analysis among clinical characteristics and laboratory variables indicated that GD (*p*<0.001) was an independent prognostic factor to predict PFS and OS. This study further confirmed the efficiency of using GD as an effective predictor of PFS (**Table 3**) and OS in HCC (**Table 4**).

**Table 2:** Univariate analyses of variables associated with PFS and OS.

	PFS		OS	
Characteristic	HR (95% CI)	p-value*	HR (95% CI)	p-value*
Age (years)				
≥60.00				
<60.00	1.09 (0.80–1.47)	0.596	1.53 (1.06–2.22)	0.025
Gender				
Female				
Male	0.72 (0.51–1.01)	0.058	0.58 (0.40–0.85)	0.005
Stage				
I				
II	3.18 (2.05–4.93)	<0.001	2.12 (1.20–3.75)	<0.001
III	3.47 (2.31–5.22)	<0.001	4.53 (2.71–7.58)	<0.001
IV	7.83 (2.71–22.59)	<0.001	29.89 (9.65–92.53)	<0.001
Tumor thrombus				
without				
With	1.80 (1.31–2.45)	<0.001	2.11 (1.57–3.09)	<0.001
GD^1^				
<43.00				
43.00–200.00	1.98 (1.38–2.83)	<0.001	3.74 (2.44–5.71)	<0.001
≥200.00	3.98 (2.69–5.90)	<0.001	5.98 (3.77–9.47)	<0.001
AFP (ng/ml)				
<20.00				
≥20.00	2.31 (1.70–3.14)	<0.001	2.29 (1.59–3.30)	<0.001
Size (cm)				
<2.00				
2.00–5.00	1.35 (0.89–2.04)	0.157	2.88 (1.62–5.12)	<0.001
5.00–10.00	2.08 (1.39–3.12)	<0.001	4.15 (2.337–7.29)	<0.001
≥10.00	2.54 (1.39–4.64)	0.002	6.51 (3.08–13.75)	<0.001
Metastasis				
Without				
With	3.12 (1.99–4.89)	<0.001	2.97 (1.67–5.29)	<0.001
Targeted Therapy				
Without				
With	0.92 (0.67–1.26)	0.606	0.98 (0.68–1.42)	0.904
Immunotherapy				
Without				
With	1.03 (0.72–1.46)	0.871	1.39 (0.94–2.05)	0.105

*P-values were calculated and based on training dataset.

1GD group for PFS was low risk group(<28.00), median risk group(28.00-160.00) and high risk group(>160.00).

**Table 3:** Multivariate Cox regression analyses of variables associated with PFS.

Characteristic	HR	95% CI	p-value
Stage			
I			
II	3.04	2.07–4.48	<0.001
III	2.88	1.86–4.43	<0.001
IV	4.17	1.39–12.51	0.011
GD			
<28.00			
28.00–160.00	1.79	1.32–2.42	<0.001
>160.00	3.61	2.34–5.56	<0.001
AFP (ng/ml)			
<20.00			
≥20.00	1.73	1.34–2.23	<0.001
Targeted therapy			
Without			
With	0.45	0.34–0.60	<0.001

**Table 4:** Multivariate Cox regression analyses of variables associated with OS.

Characteristic	HR	95% CI	p-value
Stage			
I			
II	1.32	0.78–2.21	0.299
III	2.79	1.66–4.70	<0.001
IV	16.17	5.10–51.29	<0.001
GD			
<43.00			
43.00–200.00	2.02	1.41–2.90	<0.001
≥200.00	3.14	2.09–4.71	<0.001
AFP(ng/ml)			
<20.00			
≥20.00	1.9	1.39–2.60	<0.001
Targeted therapy			
Without			
With	0.7	0.50–0.98	0.037

### 3.5 Visualization and assessment of the predictive model

**Figure 3:**
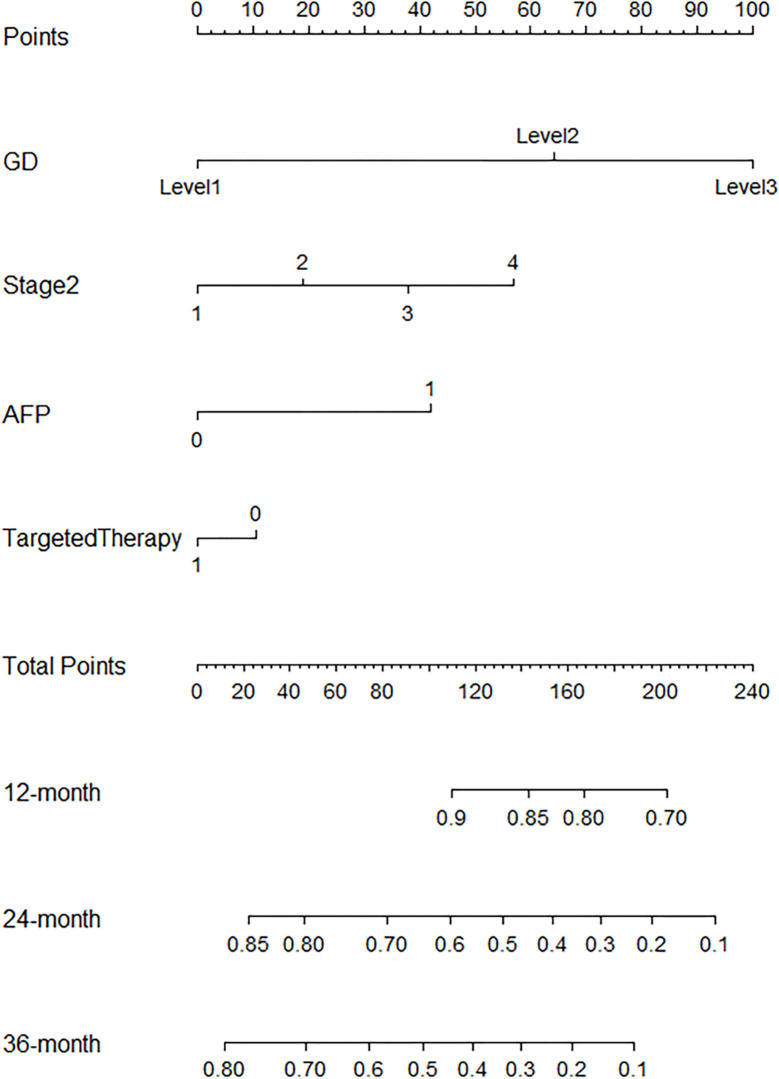
Nomogram is built to predict the overall survival.

**Figure 4:**
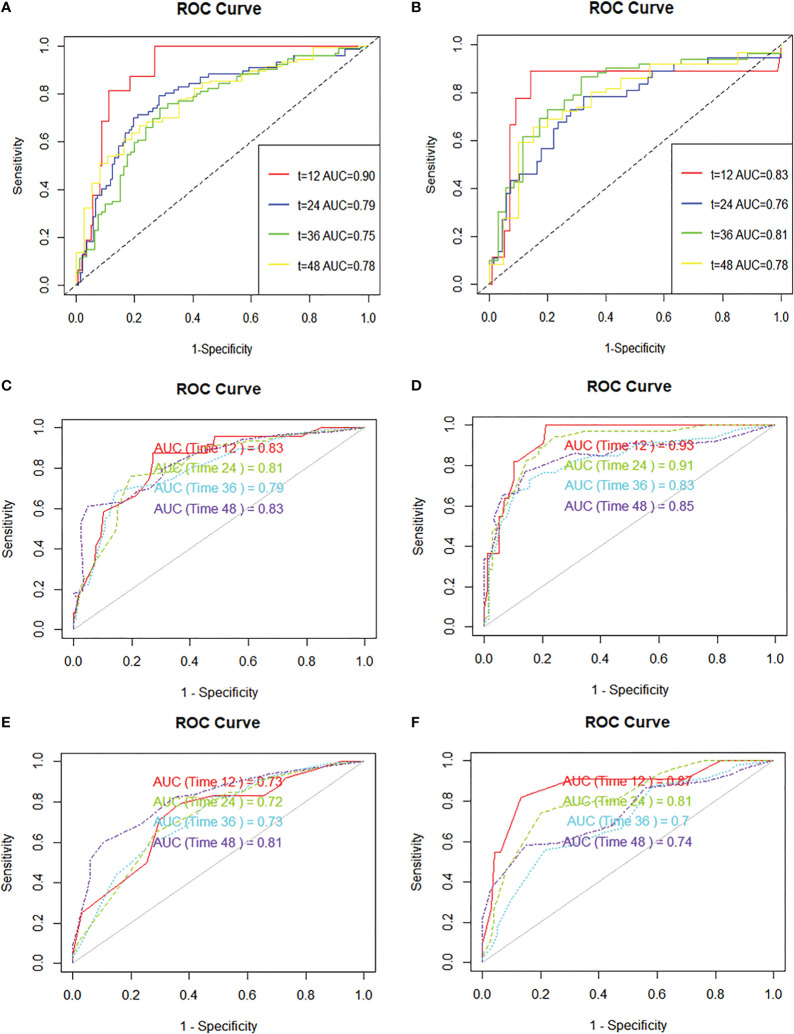
ROC curves are plotted based on different markers. **(A)** ROC curve and AUC of GD variable alone (continuous variables) in the training cohort, **(B)** ROC curve and AUC of GD variable alone (continuous variables) in the validation cohort, **(C)** ROC curve and AUC of the nomogram to predict 1-, 2-, 3-, and 4-year overall survival in the training cohort, **(D)** ROC curve and AUC of the nomogram to predict 1-, 2-, 3-, and 4-year overall survival in the validation cohort, **(E)** ROC curve and AUC of the GD-excluded cox model to predict 1-, 2-, 3-, and 4-year overall survival in the training cohort, **(F)** ROC curve and AUC of the GD-excluded cox model to predict 1-, 2-, 3-, and 4-year overall survival in the validation cohort.

R.4.1.3 nomogram package was adopted to visualize the fitted multivariate COX regression model (**Supplementary Figure S3**; **Figure 3**). A predictive model including GD, stage, targeted therapy, and AFP expression was fitted, which was confirmed to be statistically significant in multivariate COX regression analysis. The AUCs of 1-, 2-, 3-, and 4-year OS associated with GD variable alone were 0.90, 0.79, 0.76, and 0.78, respectively, in the training cohort, while that of the validation cohort were 0.83, 0.76, 0.81, and 0.78 (**Figures 4A, B**). The AUCs of 1-, 2-, 3-, and 4-year OS associated with nomogram were 0.83, 0.81, 0.79, and 0.83, respectively, in the training cohort while that of the validation cohort were 0.93, 0.91, 0.83, and 0.85 (**Figures 4C, D**). The AUCs of 1-, 2-, 3-, and 4-year OS associated with GD-excluded model were 0.73, 0.72, 0.73, and 0.81, respectively, in the training cohort while that of the validation cohort were 0.87, 0.81, 0.70, and 0.74, respectively (**Figures 4E, F**). The C-indexes of nomogram after 1,000 times of bootstraps were 0.77 and 0.76 in the training or validation cohort, respectively. The C-index of this model was greater than that of the model without GD (**Supplementary Figures S4A, B**). Consistently, the calibration curves after 1,000 times of bootstraps presented excellent uniformity of predicted and observed survival outcomes (**Supplementary Figures S4C, D**). The results above exhibited distinguished accuracy of nomogram in the prediction of OS. DCA curves analysis for the model indicated that nomogram had a greater overall net benefit than the model without GD (**Supplementary Figures S4E, F**). Additionally, absolute NRI values were calculated with 1,000 times of bootstraps, and absolute NRI values of nomogram with GD included were found to be greater than that with GD excluded (6-month PFS: NRI = 0.26, 95% CI = 0.03–0.34; 8-month PFS: NRI = 0.10, 95% CI = 0.02–0.34; 10-month PFS: NRI = 0.10, 95% CI = −0.06–0.27) (**Supplementary Table S1**), indicating the better discrimination potential of nomogram with GD than that without GD. Meanwhile, the IDI value of nomogram based on GD was 0.07 (95% CI = 0.02–0.11, p=0.01).

## 4 Discussion

A novel predictive model based on GD has been proposed in our study, demonstrating excellent predictive ability. It has been internally cross-validated through various tests including NRI, DCA curve, calibration curve, and C-index. The nomogram in our study reveals that GD, a novel indicator, exhibits prognostic ability. The AUCs of GD and the nomogram indicate their exceptional predictive power. However, it is worth mentioning that the number of events are general small for AUC, which could affect the precision and robustness of our findings. This limitation suggests that our model’s predictive ability should be interpreted with caution, and further validation in larger cohorts is warranted. In comparison to other prognostic analyses, our model incorporates three important factors: Barcelona stage, AFP, and targeted therapy. These factors play a significant role in the treatment and prognostic analysis of hepatocellular carcinoma. The inclusion of these interventions, which independently influence outcomes, not only enhances the predictive power of the model but also ensures its applicability across a broader context, making our study more comprehensive.

In this study, the significance of novel prognostic index in patients with hepatocellular carcinoma (HCC) was highlighted. Actually, these laboratory biomarkers are usually the routine check-up items for hospital examination of patients with hepatocellular carcinoma and are thus easily accessible and endowed with considerable practical clinical potential. In the first place, GD was hereby demonstrated as an independent factor that might influence the prognosis of HCC patients, and GD had remarkable significance for the prediction of recurrence or progression in most HCC patients. GGT is located on the cell membrane and catalyzes the degradation of extracellular GSH into cysteinylglycine and cysteine, which are utilized for intracellular GSH synthesis (17, 18). GGT possesses redox properties and can regulate the oxidative–reductive balance of intracellular and extracellular environment (15). Numerous studies have found elevated expression of GGT in various tumors, and high levels of GGT indicate poor prognosis. Basic research has revealed that tumor cells elevate intracellular GSH levels by overexpressing GGT, providing selective growth advantages for tumor cells and promoting tumor genesis (19). GGT maintains intracellular GSH levels, thereby countering compound toxicity, which also explains the resistance of GGT-expressing tumors to oxidants and alkylating agents (20, 21). Elevated levels of D-dimer, a degradation product of fibrin, were observed in many cancers as well, including gastric (22), colorectal (23), lung (24), ovarian (25) and prostate (26) cancers. Plasma D-dimer levels had also been found to be associated with tumor prognosis in many studies. Research has shown that the upregulation of D-dimer might be associated with tumor deterioration and shorter survival periods. In hepatocellular carcinoma, its upregulation may be related to microvascular invasion and portal vein invasion. Elevated D-dimer levels facilitate the activation of the coagulation pathway, which may promote the activation of the PI3K pathway and MAPK pathway (27), thereby stimulating tumor proliferation and invasion. GGT is associated with oxidative stress and has implications for tumor progression, while elevated D-dimer levels reflect a procoagulant and inflammatory state commonly seen in cancer patients. Combining these two biomarkers into the GD index represents a biologically plausible approach to capturing the complex interplay between oxidative stress, coagulation, and cancer progression. Furthermore, the feasibility of measuring GGT and D-dimer levels in clinical practice adds to the scientific viability of the GD index. Both GGT and D-dimer assays are routine laboratory tests and are readily available in clinical settings, making the GD index a practical and translatable parameter for patient risk stratification. By considering both GGT and D-dimer within a single index, we aim to provide a more comprehensive assessment of the tumor microenvironment and its impact on disease progression. Statistical analyses were conducted to assess the GD index’s significance in predicting patient prognosis. We used well-established statistical methods, including Kaplan–Meier survival analysis and Cox regression analysis, to evaluate the association between GD levels and patient outcomes such as PFS and OS. The results demonstrated that the GD index had a highly significant predictive value (p < 0.001), indicating its statistical significance in stratifying patients based on their prognosis. In addition, many studies have illustrated that the prognosis of HCC patients with a high AFP expression level is worse than that of patients with a low AFP expression level. AFP expression is reported to be involved in a few signal transduction, including the Wnt signaling pathway, the mitogen-activated protein kinase pathway and RA-RAR signaling pathway (27). In our study, it was also discovered that AFP expression level could be used as an independent factor differentiating the prognosis of HCC patients. In addition, nomogram is a convenient and visual tool, which is widely used in disease diagnosis and prognosis. The total point of the nomogram reflexes the probabilities of occurrence according to the scale on a ruler. Additionally, they are not only convenient and economical but also have outstanding clinical value. Neoplasm staging and therapy are major prognostic factors in many cancers, including HCC, so that a more accurate prediction could be made with these indicators taken into account. For the sake of a more accurate model and to achieve a better classification, targeted therapy, AFP expression levels and tumor stage were eventually included in the nomogram here. The subsequent evaluation also revealed the brilliant predictive ability and generalization ability of the nomogram.

However, there are still a few limitations in this study. First, this study is retrospective, failing to completely rule out the impact of selection bias, and with only Chinese subjects, which may not be applicable to other ethnic groups. Second, basic experimental verification was not carried out, so the mechanism of these laboratory indicators predicting the prognosis of patients with hepatocellular carcinoma remains unclear. Nevertheless, the findings of the present study still contribute considerably to the understanding of the relationship between tumor microenvironment and clinical factors. Additionally, larger and more detailed prospective studies are needed to further clarify these relationships.

## 5 Conclusion

Herein, GD was demonstrated as an independent prognostic factor for hepatocellular carcinoma and presented the potential to predict the PFS and OS in patients with HCC. Moreover, patients with a lower level GD had a longer PFS and OS than those with a higher level GD. A prediction model was established based on GD. In order to make the prediction model more accurate, AFP, Barcelona staging and target therapy were added, and the nomogram involving these indicators presented satisfactory prediction ability compared with other models without GD, further indicating its potential as a clinical tool to predict the PFS and OS in patients with HCC.

## Data availability statement

The datasets generated and analyzed during the current study are available from the corresponding author upon reasonable request.

## Ethics statement

The studies involving humans were approved by Ethics Committee of The First Affiliated Hospital of Zhengzhou University (approval number: 2022-KY-1140). The studies were conducted in accordance with the local legislation and institutional requirements. The participants provided their written informed consent to participate in this study.

## Author contributions

Conceptualization, KC. Methodology, YL. Software, YL. Data curation, YL. Writing—original draft preparation, YL. Writing—review and editing, HZ. Visualization, YL. Supervision, WM. All authors have read and agreed to the published version of the manuscript.
