# Supplementary figures and images for: A novel nomogram based on GD for predicting prognosis in hepatocellular carcinoma
Source: Front Oncol. 2023 Nov 1;13:1174788. doi: 10.3389/fonc.2023.1174788 (PMC10646613; doi:10.3389/fonc.2023.1174788)

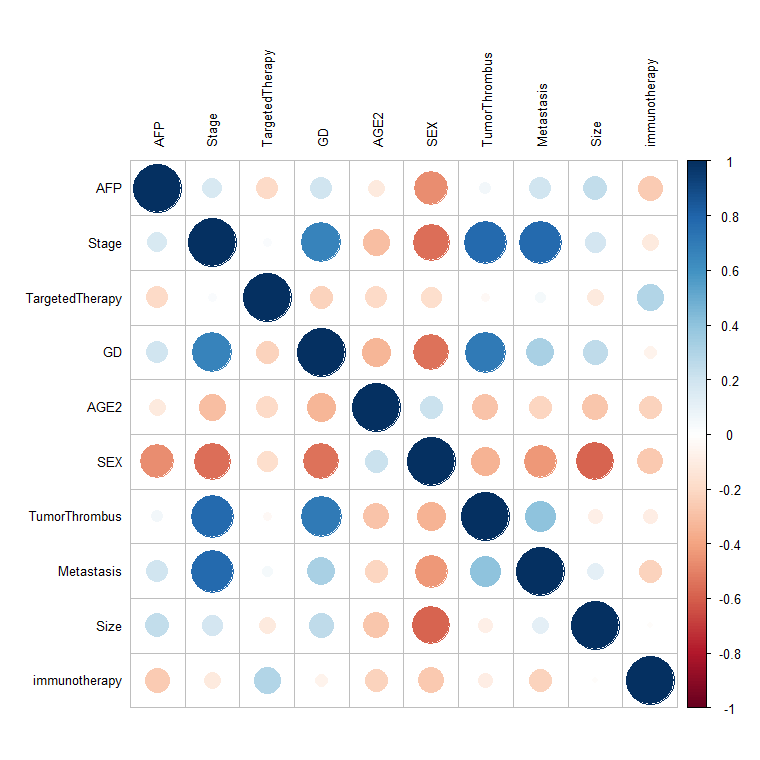

Supplement: Supplementary file 1 [file Image_1.tif]

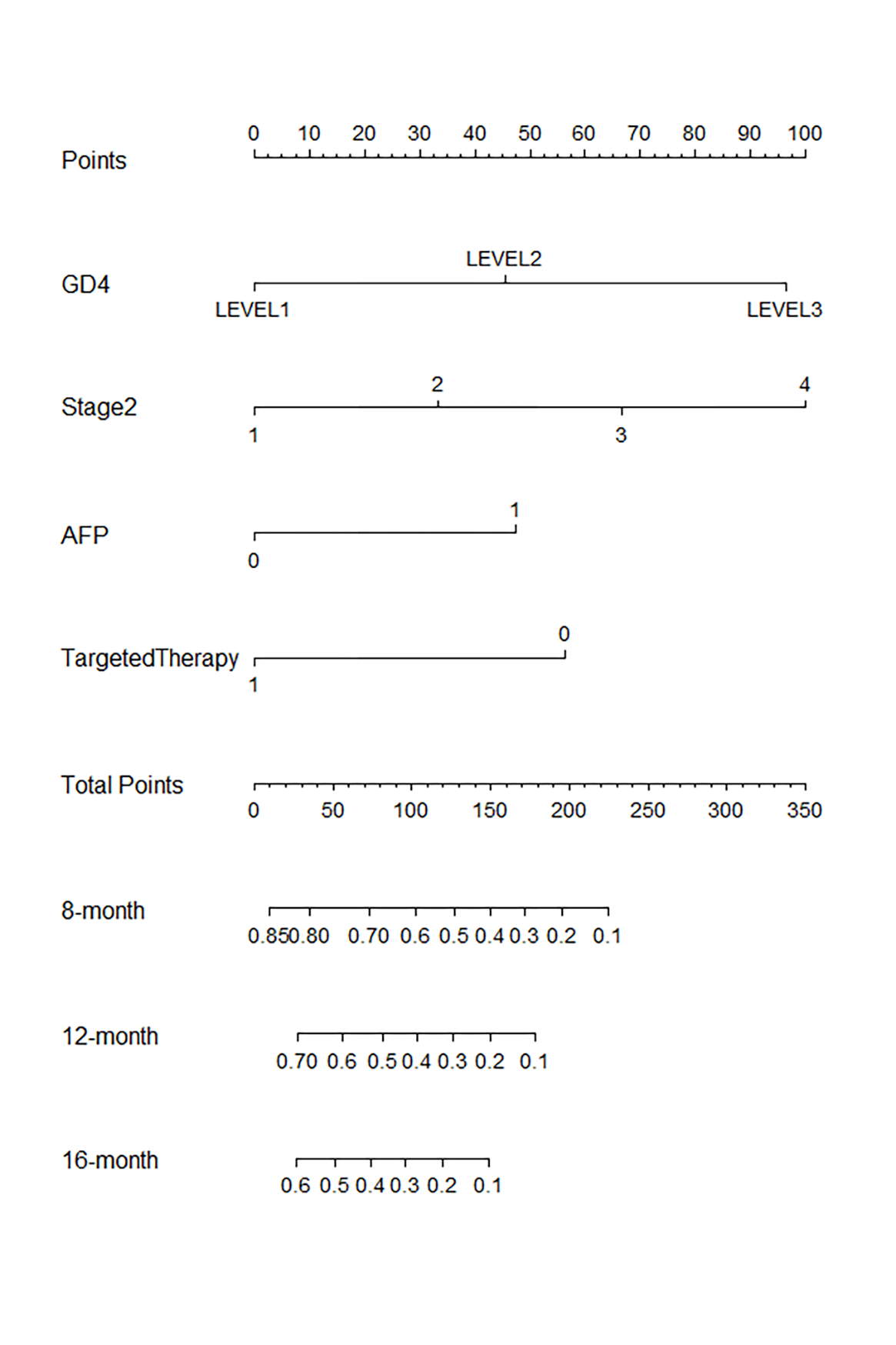

Supplement: Supplementary file 2 [file Image_2.tif]

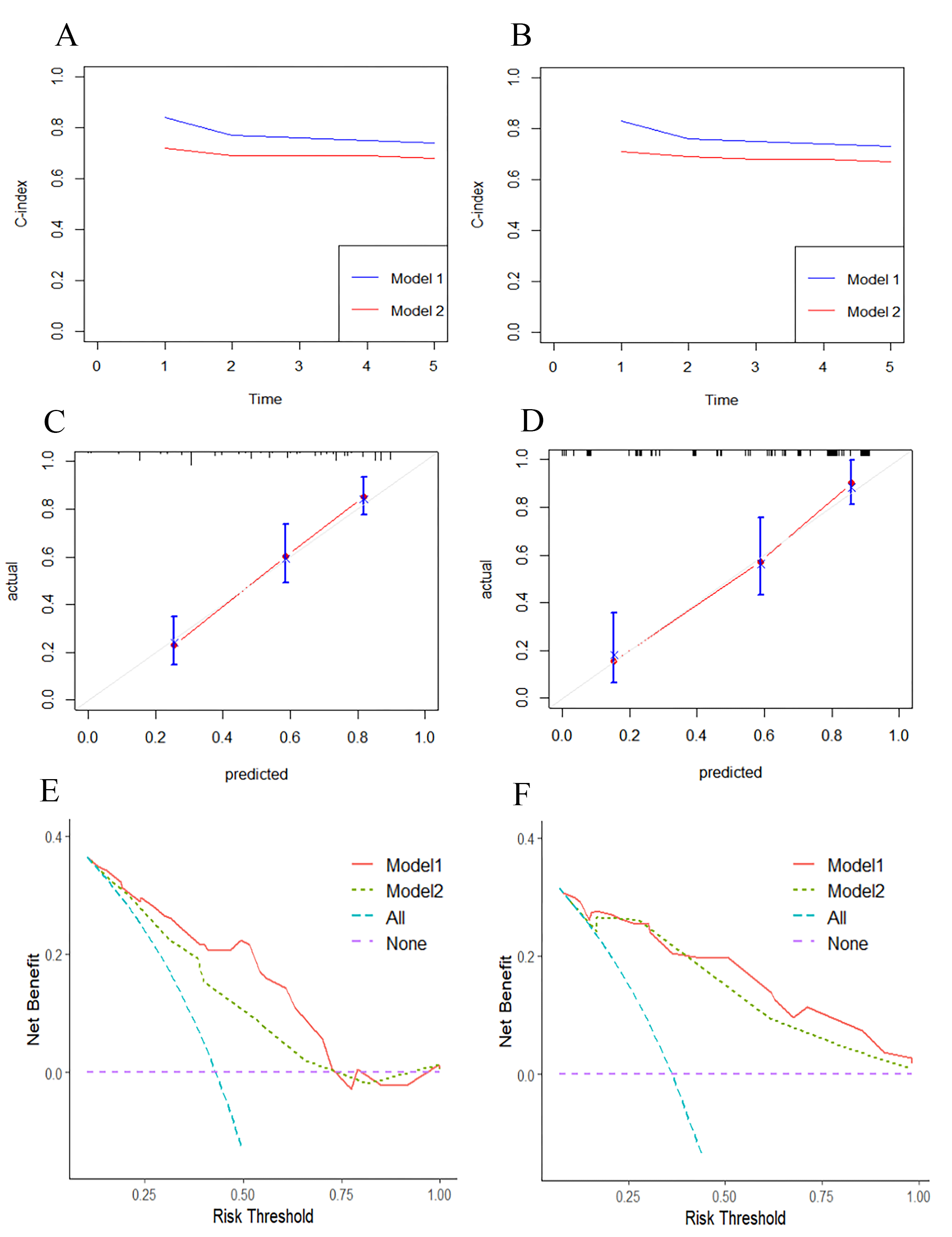

Supplement: Supplementary file 3 [file Image_3.tif]
