# Supplementary material for: A novel nomogram based on GD for predicting prognosis in hepatocellular carcinoma
Source: Front Oncol. 2023 Nov 1;13:1174788. doi: 10.3389/fonc.2023.1174788 (PMC10646613; doi:10.3389/fonc.2023.1174788)
Supplement: Supplementary file 4 [file DataSheet_1.docx]

**Table S1**

NRI of predictive model with GD

| **PFS** | **NRI(95%CI)** |
| --- | --- |
| 6-month | 0.262(0.030– 0.340) |
| 8-month | 0.104(0.017–0.339) |
| 10-month | 0.102(-0.059–0.268) |

**Figure S1** Consort diagram

**Figure S2** The correlation among 10 variables.

**Abbreviation** Stage, Barcelona stage; Throm, Tumor Thrombus; Met, metastasis; Treatment, Targeted Therapy; LNM, lymph node metastasis

**Figure S2** Nomogram is build to predict the progression-free survival.

**Figure S3** assessment of the nomogram.

**A**.C-Index of the nomogram in the training cohort, **B**.C-Index of the nomogram in the validation cohort,（C-index1, C-index of predictive model with GD, C-index2, C-index of predictive model without GD). **C.** the calibration curves of the nomogram in the training cohort, **D.** the calibration curves of the nomogram in the validation cohort, **E.** The DCA curve of the nomogram in the training cohort, **F.** The DCA curve of the nomogram in the validation cohort,（model1,predictive model with GD; model2, predictive model without GD).
